# *In vitro* and *in vivo* evaluation of orthopedic interface repair using a tissue scaffold with a continuous hard tissue-soft tissue transition

**DOI:** 10.1186/1749-799X-8-18

**Published:** 2013-06-19

**Authors:** Darryl A Dickerson, Tarik N Misk, David C Van Sickle, Gert J Breur, Eric A Nauman

**Affiliations:** 1 Weldon School of Biomedical Engineering, Purdue University, West Lafayette, IN, 47907, USA; 2 School of Veterinary Medicine, Purdue University, West Lafayette, IN, 47907, USA; 3 Department of Basic Medical Sciences, Purdue University, West Lafayette, IN, 47907, USA; 4 School of Mechanical Engineering, Purdue University, 585 Purdue Mall, West Lafayette, IN, 47907-2088, USA

**Keywords:** Tendon repair, Soft tissue-bone interface, Rotator cuff tenotemy

## Abstract

Tendon tears produce pain and decrease joint stability; each year, over 1.1 million rotator cuff tendon surgical procedures are performed worldwide. However, surgical success is highly variable, and the inability of the procedure to drive the regeneration of the normal tendon-bone interface has been identified as a key factor in surgical failure. This study focuses on the development, *in vitro* evaluation, and *in vivo* assessment of a tissue scaffold derived from bovine cancellous bone with the potential to direct regeneration of a bone-soft tissue interface. The scaffold is a highly porous scaffold with a continuous hard tissue-soft tissue transition that facilitates load transfer across the interface and contains all of the extracellular matrix components of the orthopedic interface. This study demonstrated the *in vitro* characterization of the mechanical properties and successful *in vivo* assessment using an ovine model.

## Introduction

Rotator cuff injuries in the shoulder are common, with more than 17 million people in USA having some form of rotator cuff tendon tear [1]. Tendon tears are painful, can decrease joint stability, have limited healing potential, and often lead to arthritis [2,3]. To prevent irreversible shoulder joint damage, each year, over 1.1 million rotator cuff tendon surgical procedures are performed worldwide. However, surgical success is highly variable with failure rates ranging from 30% to 90%, depending on the severity of the tendon defect and the stage of degeneration of the joint [4,5]. The inability of surgical procedures to drive the regeneration of the normal tendon-bone interface has been identified as a key factor in surgical failure [6].

Prior studies using tendinous autografts have shown that using the midsubstance as the repair material does not lead to strong biological integration or the re-establishment of the native orthopedic interface structure [7,8]. In order to restore the physiological structure and function of the tissue, new strategies must be developed for the treatment of soft connective tissue ruptures. A number of different strategies have been employed, including applications of cells [9,10], growth factors [11,12], gene therapy products [13,14], and biomaterial scaffolds [15-18]. While each of these approaches has their merit, practically speaking, the use of cells, growth factors, and gene products have significant limitations currently. The use of cells requires an additional surgical procedure that may produce unnecessary morbidity [19-22]. Soluble growth factors and gene therapy are limited by inadequate means of delivery to the target site and unknown specificity [11,23]. Because the use of non-autologous cells, growth factors, and gene therapy require considerable additional regulatory hurdles, the most robust solution may lie in the use of biomaterial scaffolds to guide the regeneration of the tendon-bone interface.

Additionally, since many of these scaffolds are produced to mimic only the soft tissue midsubstance, anchoring the structure to the bone presents additional challenges. Consequently, we propose the following design requirements intended to optimize the development of a tissue-engineered solution for soft connective tissue rupture. First, the design requires a biocompatible scaffold that has the mechanical properties to withstand the loading environment during recovery. While this does not necessarily mean that the scaffold must be fully load-bearing immediately after surgery, its failure strain should be similar to or greater than the strain that would be expected during normal range of motion. The scaffold must also have a high fluid conductance, concomitant with high porosity, as demonstrated by Hui et al. [24] in a landmark study. Porosity allows more rapid cell incorporation along the surface and through the thickness of the scaffold, promoting integration with the host tissue. Additionally, porosity allows early vascular ingrowth so that the metabolic demands of the new tissues can be met, ensuring cell viability throughout its thickness [24]. While the critical level of transport has not been determined for the bulk of orthopedic tissues, it is clear that mechanical strength and high porosity are competing objectives and each must be carefully balanced within the design. Tissue ingrowth is vital, but, specifically for this application, it must be accompanied by biological integration, so that the normal interface is reformed. Next, cell behavior is expressly controlled by the interaction with its extracellular environment, in particular the biomaterial surface [25-27]. The scaffold must guide cells to regenerate all four zones of the tissue structure.

Developing a contiguous biomaterial to meet these design requirements presents a significant challenge. The control that the biomaterial surface must exert on the cellular response requires heterogeneity of the structural scaffold in order to reproduce the heterogeneity of the normal interface while maintaining continuous load transfer. We propose the development of such a composite structure derived from cancellous bone to serve as the scaffold for orthopedic interface regeneration. This study focuses on the development, *in vitro* evaluation, and *in vivo* assessment of a tissue scaffold derived from bovine cancellous bone with the potential to direct regeneration of a bone-soft tissue interface. Cancellous bone has high porosity [28] to permit transport and can be obtained from anatomic sites that exhibit highly directional trabeculae to promote load transfer from the bone to the tendon. We hypothesized that when augmented with this tissue scaffold and contacting the marrow space, the tendon-bone interface would be substantially improved compared to standard primary repair.

## Materials and methods

### Cancellous bone preparation

Samples were prepared from bovine cancellous bone harvested from the vertebral bodies of commercially obtained steers (Parrett’s Meat Processing, Flora, IN). Residual soft tissue was dissected from the vertebral bodies. Using a diamond saw, the cancellous interiors of the vertebrae were sectioned into 3 mm × 3 mm × 40 mm blocks, the long axis aligned with the antero-posterior direction of the vertebral column. These samples were used for mechanical testing. Small scaffolds for cell culture studies were similarly machined to a size of 3 mm × 3 mm × 2 mm. Cylindrical samples for micro-computed tomography (μCT) imaging were obtained from 5-mm sections of the vertebral body using a 6-mm hollow punch. For surgical implantation, cores 5 mm in diameter and 20 mm in length were machined from the vertebral body. All samples were washed in a 1% detergent solution (Tergazyme®, Alconox, White Plains, NY) to remove fat and marrow from the trabecular space. Samples were then thoroughly washed in deionized water for 4 h and defatted by soaking in 100% acetone for 12 h.

### Determination of minimum demineralization time

Small scaffolds (*n* = 6) approximately 3 × 3 × 2 mm were placed in a volume (50 mL per gram bone) of demineralizing solution (1.0 M HCl, 1.9 mM ethylene diamine tetra-acetic acid) for 0, 1, 4.5, and 24 h to determine the minimum amount of time needed to fully demineralize the samples. Following demineralization, the samples were thoroughly washed in deionized water. The scaffolds were dried at room temperature for 24 h and weighed using an analytical balance. Each scaffold was placed into 6 mL of 2 M HCl for 24 h to release all the calcium. Each sample's calcium content was measured using inductively coupled plasma atomic emission spectroscopy (ICP-AES). ICP-AES is a high sensitivity method for measuring trace elements in solution. After nebulizing the samples, the aerosol flows through a plasma torch which generates a light spectrum. The resulting spectra can be analyzed to determine the mass of calcium present in the sample.

### Mineral protection testing

Small undemineralized disks (*n* = 6) approximately 6-mm wide and 5-mm thick were covered with a polymer solution (nitrocellulose, New-Skin Liquid Bandage, MedTech Laboratories, Irvington, NJ) and allowed to dry. The samples were then placed in the aforementioned demineralizing solution for the previously determined minimum demineralization time and rinsed thoroughly in deionized water. The samples were analyzed before and after they were placed in the demineralizing solution using μCT to view image scaffold structures and quantify mineral content. Images were taken using a μCT imaging system (Scanco Medical μCT 40, Bassersdorf, Switzerland) that provided an isotropic resolution of 16 μm (40 kVp, integration time 200 ms). The bone volume output after three-dimensional reconstruction was used to assess mineral content with a resolution better than 0.05 g/cm^3^[29]. Because the critical diffusion length scale is the thickness of a trabecula, it is anticipated that these results will be applicable to larger scaffolds with similar trabecular structures.

### Cell extraction and culture

Human adult adipose stem cells (ASCs) were a generous gift from Dr. Keith L. March at the Indiana Center for Vascular Biology and Medicine. These cells were extracted from subcutaneous adipose tissue obtained during an elective lipoaspiration procedure as previously described by Cai et al. [30]. These cells were cultured in endothelial growth medium (Lonza, Walkersille, MD), a specialized growth medium consisting of endothelial basal medium-2 (Lonza), 5% fetal bovine serum (FBS), and supplemental growth factors including vascular endothelial growth factor, basic fibroblast growth factor, epidermal growth factor, and insulin-like growth factor.

### Removal of osteoinductivity

In order to successfully use demineralized bone in soft tissue regeneration applications, it is important to remove the inherent osteoinductivity, a process that has been previously accomplished through the use of hydrogen peroxide [31,32]. To determine the effect of peroxide treatment on cell-matrix interactions, cell attachment and proliferation were assessed on untreated and peroxide-treated scaffolds (*n* = 6/group). Both untreated and peroxide-treated scaffolds were then thoroughly rinsed in deionized water and sterilized with 70% ethanol overnight. Sterile scaffolds were placed into 24-well plates and suspended in a cell solution containing 10^5^ ASCs. The cells were allowed to settle onto the scaffold for 12 h in a humidified 37°C, 5% CO_2_ incubator. After this period, the scaffolds were transferred to another 24-well plate. Full media exchange was performed every other day. The scaffolds were cultured for 1 day to assess cell attachment and 5 days to assess cell proliferation. At these time points, culture medium was removed from the wells. The scaffolds were covered with a 0.9% solution of Triton X-100 in order to release the intracellular content. CytoTox 96 non-radioactive cytotoxicity assay (Promega Corporation, Madison, WI) was used to measure lactate dehydrogenase (LDH) activity. The total LDH activity was compared across groups to obtain a relative measure of cell number.

Two methods were used to assess the efficacy of peroxide in reducing osteoinductivity. The first method made use of alkaline phosphatase (ALP) activity as a measure of osteogenic differentiation of ASCs [33]. Untreated and peroxide-treated scaffolds were fragmented into powder form in a liquid nitrogen impacting mill. The untreated and peroxide-treated powders were weighed and then sterilized in 70% ethanol overnight. The powders were then suspended at 10 mg/mL in Dulbecco's modified eagle medium (DMEM, Invitrogen, Carlsbad, CA) supplemented with 10% (Invitrogen), 50 μg/mL ascorbic acid (Sigma-Aldrich, St. Louis, MO), and 10 mM *β*-glycerophosphate (Sigma-Aldrich). Into each well of a 48-well plate, 10^5^ ASCs were transferred. After a 5-h attachment period, medium with untreated powder, medium with peroxide-treated powder, or medium without powder was added to each of the wells. Full media exchange was performed every other day. After 7 days, the cells were lysed with deionized water and three freeze-thaw cycles. ALP activity in the cell lysate was determined using the malachite green assay from a commercially available kit (Upstate Cell Signaling, Lake Placid, NY) [33]. The second method involves the assessment of the mineral deposition on the scaffolds after a long-term culture. Sterile untreated and peroxide-treated scaffolds were seeded with 10^5^ ASCs and cultured for 30 days in DMEM supplemented with FBS, 50 μg/mL ascorbic acid, and 10 mM *β*-glycerophosphate. Full medium exchange was performed every other day. After 30 days, the mineral deposition was assessed using μCT imaging.

### Structure and surface morphology

To determine the effect of peroxide treatment on the structure of the scaffold, scanning electron microscopy (SEM) analysis was performed on untreated and peroxide-treated demineralized bone cylinders (*n* = 3 per group). Each sample was gold sputter-coated (LADD, Williston, VT) in a 3.6 μtorr vacuum at 5 kV and 6 mA. The samples were analyzed using a JEOL-840 microscope (Jeol Ltd., Tokyo, Japan) imaging from ×150 to ×5,000 magnification.

### Mechanical testing

Mechanical testing samples were generated by regionally demineralizing long cancellous bone samples as briefly described here. The pores of the long cancellous structures were cleansed by alternately sonicating in a 1% detergent solution (Tergazyme®, Alconox, White Plains, NY) and rinsing with running water (Millipore, Billerica, MA). The cancellous bone cylinders were defatted by soaking in 100% acetone for 12 h. A nitrocellulose coating was applied to the 5 mm of each end of the long structure to protect this region from demineralization. The structures were placed in a demineralizing solution composed of 1.0 M hydrochloric acid and 1.9 mM ethylene diamine tetraacetic acid for minimum demineralization time as previously determined. The nitrocellulose coating was removed through a 100% acetone bath for 12 h, resulting in a biomesh with two hard regions at the end, a fully demineralized soft region, and a contiguous transition zone with a gradient of mineralization between the two regions. The samples were then treated with 3% hydrogen peroxide to remove osteoinductivity.

Long samples (*n* = 4 per group, 40-mm long) were tested in uniaxial tension to obtain load-deformation curves. The samples were hydrated in phosphate-buffered saline for at least 4 h prior to the test. Each sample was mounted between rubbers with an adhesive to ensure grip attachment. The samples were loaded into the aluminum clamps of a custom-designed mechanical testing unit consisting of a linear actuator and a 111 N load cell. The gauge length was set to be 30 mm between the clamps. The samples were pre-loaded to 0.1 N, less than 1% of the ultimate tensile load of the weakest sample. Force output and position were then set at 0 from this position. Device operation and data acquisition were controlled with LabView software (National Instruments, Austin, TX). The samples were loaded to failure at a 1 mm/s displacement rate. The curves of the nominal stress vs. Green's strain were calculated from the load-deformation output. The ultimate tensile stress, strain at failure, and tangent modulus were calculated from these curves.

### Experimental ovine rotator cuff model

Three ewes (80 to 110 kg) underwent rotator cuff defect induction and repair surgery. During a 7- to 14-day conditioning period, general physical and lameness exams, as well as a complete blood count, serum chemistry, and urine analysis were performed to ensure animal health. All animals were allowed to move freely in their pens while being monitored for post-surgical complications. The study was approved by the Purdue Animal Care and Use Committee.

**Figure 1:**
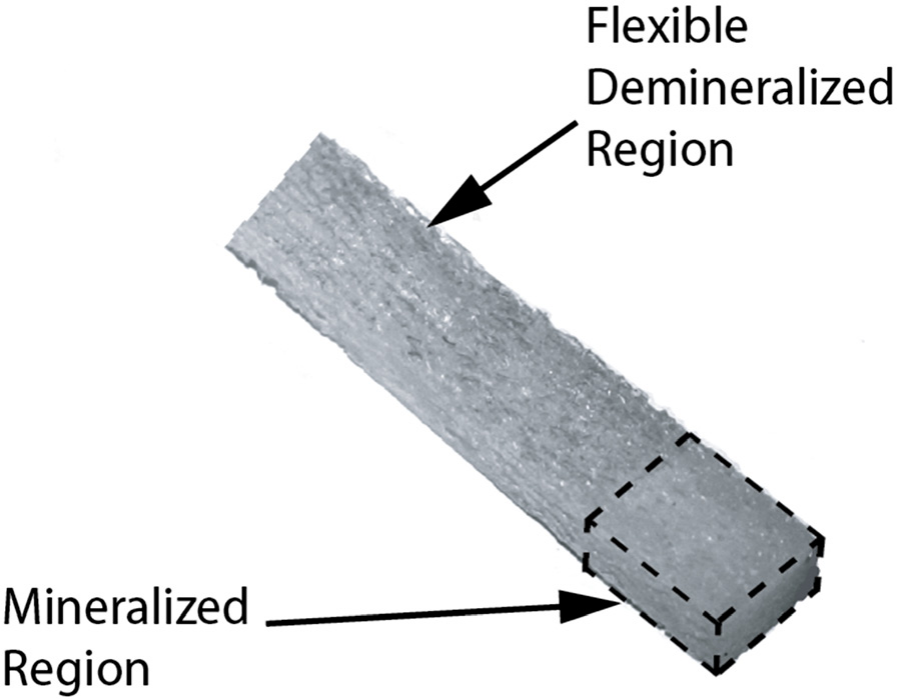
**Regionally demineralized cancellous bone scaffold.** The flexible demineralized region in the center and the hard region at the end highlighted for clarity.

The 5-mm diameter and 20-mm long cancellous bone cylinders were prepared, and after drying in air, 1 cm of one end of the cancellous bone core was placed in a solution of a polymer solution (New-Skin Liquid Bandage) and allowed to dry. The core was then placed in a volume of the demineralizing solution (1 M HCL, 1.9 mM ethylene diamine tetraacetic acid) for 4.5 h, producing the tissue scaffold with a hard tissue segment and a soft tissue segment (Figure 1). Following this demineralization bath, the scaffold was washed thoroughly with deionized water. The polymer was removed from the mineralized end with another 12-h acetone wash. After air drying, 3% hydrogen peroxide was used to remove osteoinductive factors from the scaffold. The tissue scaffold was then sterilized in 70% ethanol for 24 h, rinsed in sterile physiological saline, and stored in the saline solution until surgery.

### Surgical procedure

All animals were premedicated with diazepam (0.1 to 0.5 mg/kg intravenously or 0.2 to 1.0 mg/kg intramuscularly) followed by induction with either pentothal (12 mg/kg intravenously to effect) or a combination of ketamine (2 to 6 mg/kg intravenously) and xylazine (0.5 to 0.15 mg/kg intramuscularly or intravenously). Anesthesia was maintained with a mix of isoflurane (1% to 5%) and O_2_. Post-operative pain medication consisted of morphine (0.2 to 0.5 mg/kg intramuscularly or intravenously, one dose at closure and another at or after extubation) and buprenorphine (0.005 to 0.01 mg/kg intramuscularly every 8 to 12 h for 24 to 48 h post-surgery).

**Figure 2:**
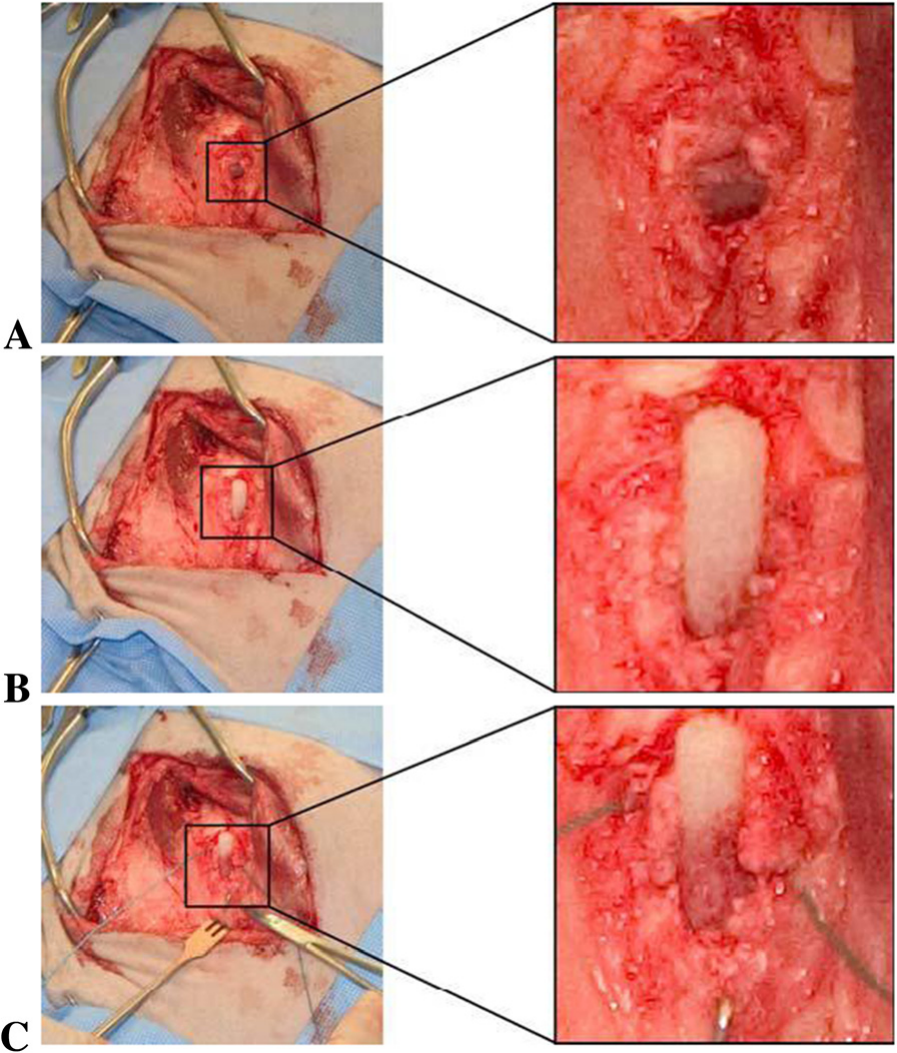
**Wicking action of the blood and marrow into the implanted scaffold.** After drilling a 5-mm hole (**A**) into the humerus, the scaffold was positioned (**B**) and pinned into the site (**C**). Over time, prior to completion of suturing, the blood and nutrients from the marrow space are wicked into the porous mesh (**C**).

Bilateral surgery was performed on three ewes by a board certified veterinary surgeon in a procedure similar to that used for previous studies [34]. The infraspinatus and supraspinatus tendon were approached by making a curved incision over the distal portion of the scapular spine continuing over the craniolateral surface of the proximal half of the humerus. An incision was then made through the brachial fascia just cranial to its attachment to the acromial part of the deltoid muscle. The deltoid muscle was retracted to expose the infraspinatus and supraspinatus tendons and muscles. The defect of the infraspinatus tendon insertion was created by making an inverted T-shaped incision in the most distal portion of the infraspinatus tendon; this incision resulted in a partial tenotomy (50% tendon, approximately 1 cm) with an intact cranial (0.5 cm) and caudal (0.5 cm) post. Then, the non-mineralized fibrocartilage of the partial tenotomy was removed, and a 5-mm hole was drilled in the exposed enthesis. This procedure was repeated in the supraspinatus tendon. The induced defects were treated either using the control treatment or the experimental treatment. Control treatment consisted of standard primary repair in which the remaining tendon portions were reconnected by suturing with a #2 Tevdek locking loop pattern and attached to the enthesis using a bone tunnel. Experimental treatment consisted of press fitting the hard portion of the tissue scaffold into the bone and stabilizing it with a small cross pin. The marrow and blood were allowed to wick up into the structure (Figure 2). The flexible segment of the tissue scaffold was sutured to the remaining portion of the tendon and muscle (suprapinatus muscle). In each shoulder, one tendon was control-treated, while the other tendon was experimentally treated. The treatment was reversed on the contralateral side. The incision was closed by suturing the brachial fascia to the acromial head of the deltoid muscle (No. 2 polydioxanone, continuous pattern) followed by closure of the subcutaneous muscle (No. 2 polydioxanone, continuous pattern) and the skin (skin staples). In total, six tissue scaffolds were implanted, and six control procedures were performed.

### Specimen retrieval and histological analysis

All animals were sacrificed at 16 weeks post-operatively by intravenous injection with an overdose of barbiturate (beuthanasia, 39 mg/kg). A necropsy was performed and the infraspinatus and supraspinatus tendon complexes were isolated and placed in 10% neutral buffered formalin for histologic examination. After fixation of the tissues at room temperature for at least 1 week, the nondecalcified specimens were embedded in plastic and sectioned longitudinally to a thickness of 50 microns through the sample to obtain a plane containing the repaired tendon defect. The sections were stained with toluidine blue for standard histological assessment and MacNeal's tetrachrome to visualize the heterogeneous structural components of the tendon-to-bone interface. Stained sections were analyzed via transmitted light microscopy with an Olympus BX51 (Olympus America Inc., Center Valley, PA, USA) microscope equipped with polarizing filters.

### Statistical analysis

Statistical analysis was conducted by repeated measures one-way analysis of variance (ANOVA) for calcium content and ALP activity, and by two-way ANOVA for cell viability data. A *post hoc* Bonferroni-Dunn test was used to test the effects of individual variables split by time where appropriate. A significant effect was defined as *p* < 0.0167.

## Results

### Minimum demineralization time

**Figure 3:**
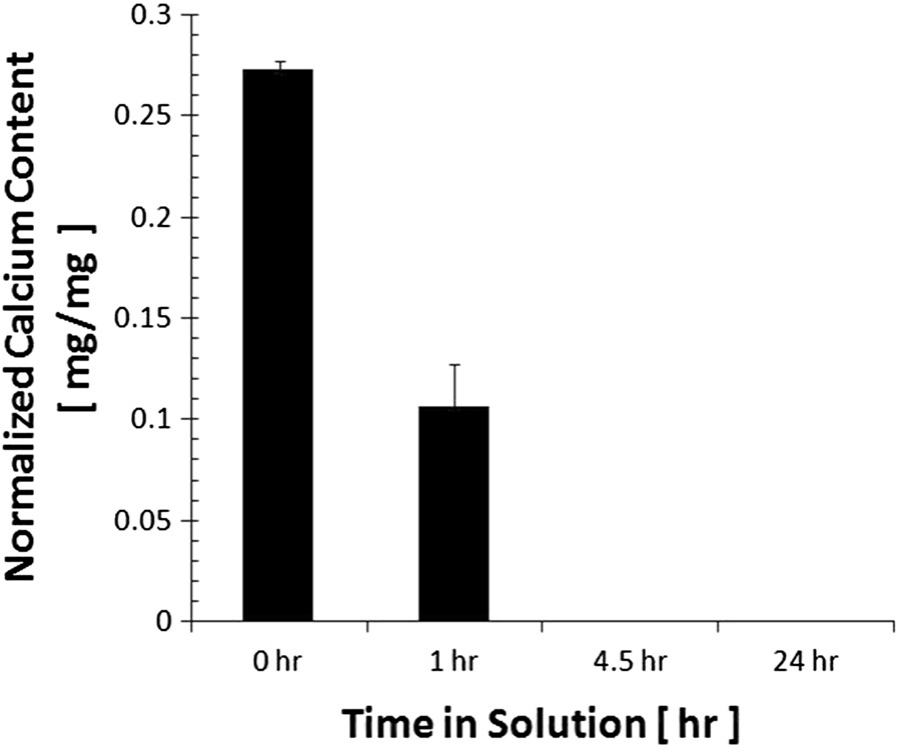
**Normalized calcium content in cancellous bone samples.** The calcium content is a function of time in demineralizing solution (*n* = 3 per group) as measured by ICP-AES. After 4.5 h, the mineral has been completely removed.

Calcium content per dry weight scaffold as determined by ICP-AES was 27.2% ± 0.4%, 10.6% ± 2.1%, 0.0% ± 0.0%, and 0.0% ± 0.0% in small scaffolds exposed to demineralizing solution for 0, 1, 4.5, and 24 h, respectively (Figure 3). Based on these data, it was determined that most of the mineral mass is lost in the first hour in the solution, and the samples are completely demineralized after only 4.5 h of treatment.

### Mineral protection

**Figure 4:**
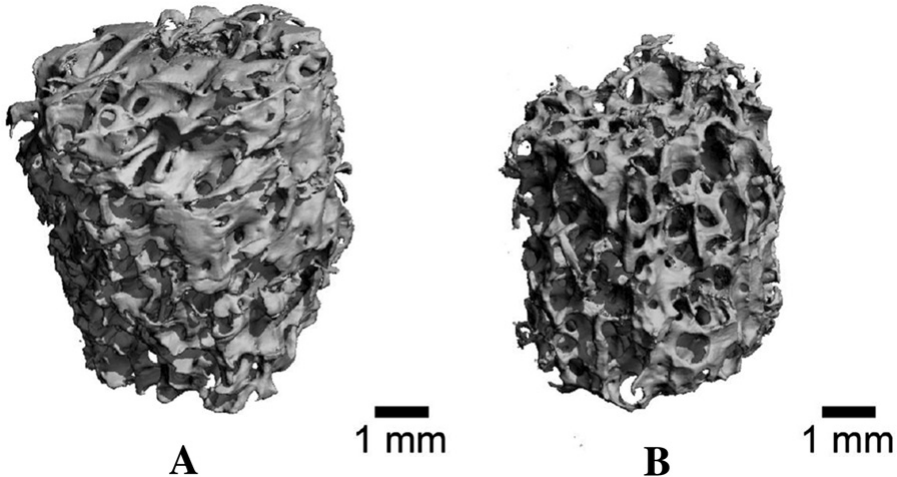
**μCT reconstruction of a small cancellous bone cylinder protected with a polymer coating.** (**A**) Before and (**B**) after exposure to the demineralizing solution for 4.5 h. The outer portions of the cancellous bone core have been demineralized, while the inner portions retain their mineral structure.

In order to create a contiguous scaffold with mineralized and demineralized regions, the samples were coated with a polymer to protect the regions from being demineralized within the minimum 4.5-h exposure time. The μCT scans were taken before and after the demineralization process to determine the efficacy of the treatment (Figure 4). The protective polymer coating was able to retain 58.0% ± 17.3% of the mineral within the scaffolds. The outer ring was substantially demineralized, leaving the inner core region mineralized.

To determine if using such a coating could produce a scaffold with mineralized and demineralized sections, the ends of the long cancellous bone samples were coated with the polymer solution and placed in demineralizing solution for a minimum treatment time. The resulting structure constituted a regionally demineralized cancellous bone scaffold (Figure 1) with a demineralized central region, two segments with an outer ring of partially demineralized structure, and an inner mineralized core region.

### Surface morphology and cell-biomaterial interaction

**Figure 5:**
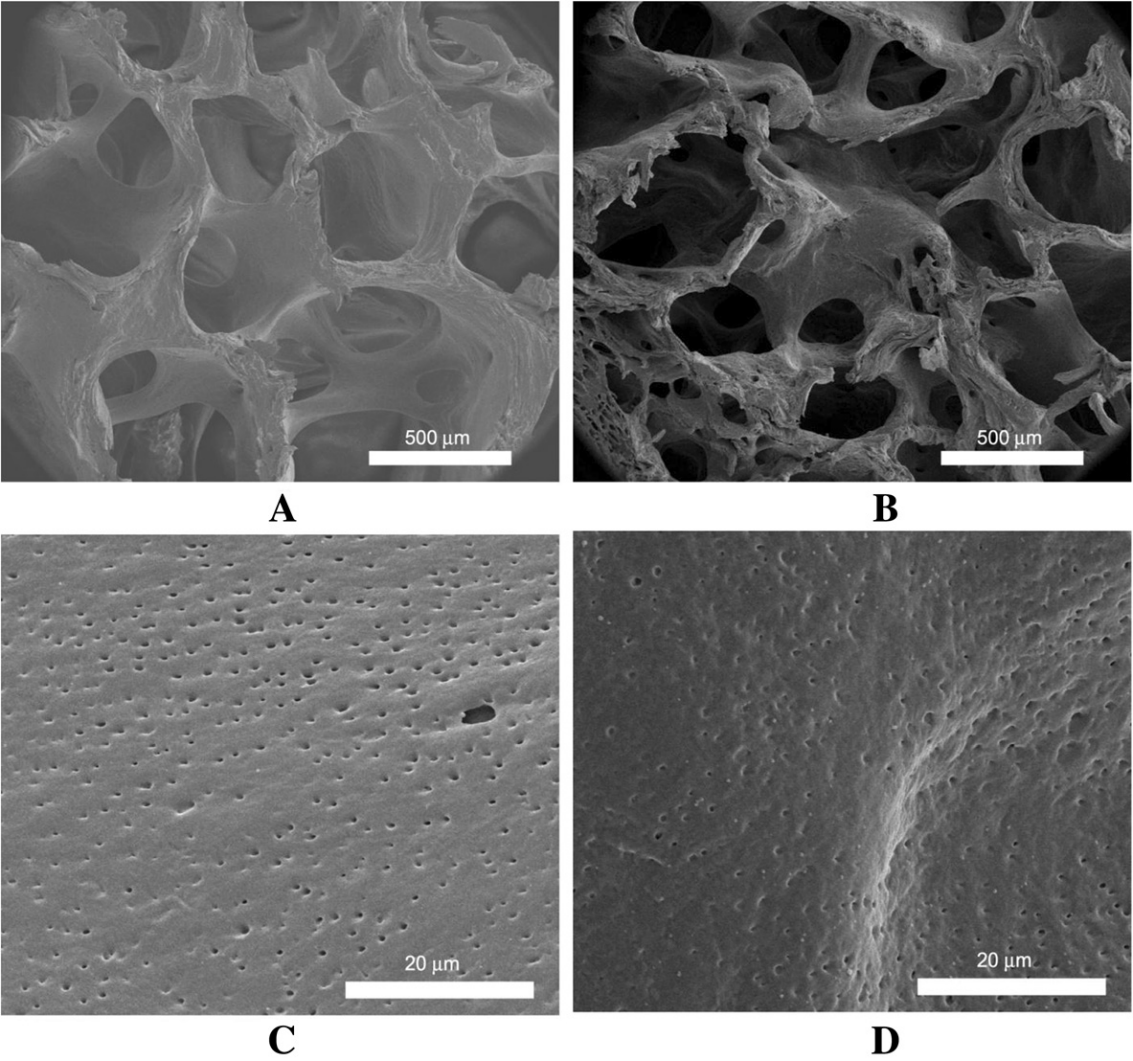
**Multiscale SEM images of untreated (A, C) and peroxide-treated (B, D) scaffolds.** The macro- and micro-scale pore structures were unchanged by peroxide treatment.

**Figure 6:**
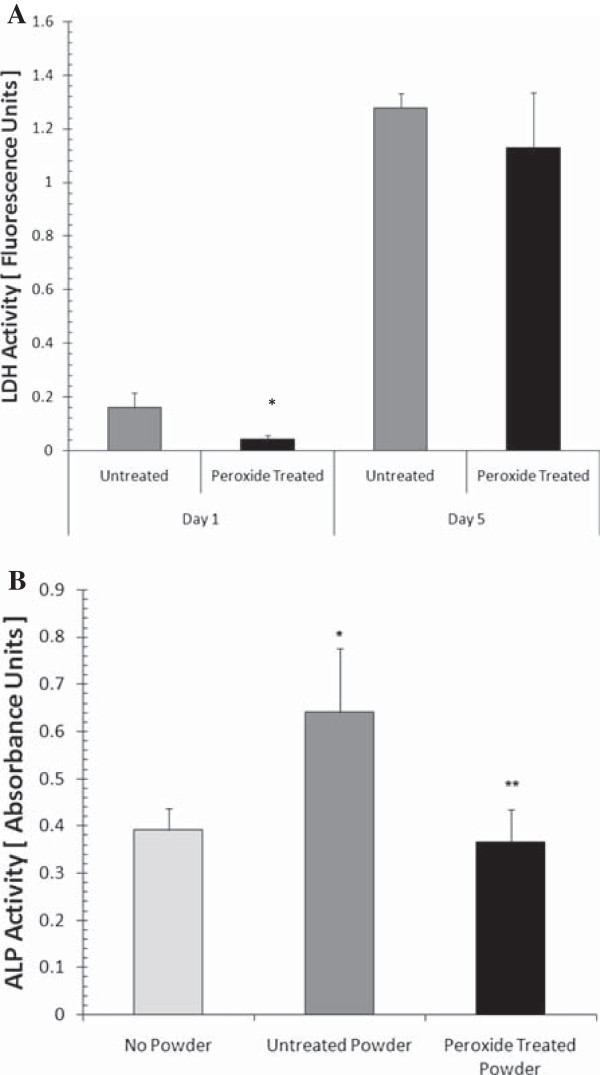
**Cell attachment and proliferation and ALP activity of monolayer ASC cells.** (**A**) Cell attachment and proliferation on untreated and peroxide-treated demineralized cancellous bone scaffolds. Cell attachment significantly decreased on peroxide-treated scaffolds compared to that on the untreated ones (denoted by asterisk, *p* < 0.05). However, the cell number was similar for the two groups after 5 days in culture. (**B**) ALP activity of monolayer ASC cells after 7 days in culture with different demineralized bone powder treatments. Statistically significant differences were found between cultures without powder and cultures supplemented with untreated powder (denoted by asterisk, *p* < 0.05), as well as cultures with untreated powder and cultures with peroxide-treated powders (denoted by double asterisks, *p* < 0.05).

Demineralized bone is a well-known osteoinductive agent. However, for this application, it is critical that the osteoinductivity of the bone be removed to promote the regeneration of soft tissue in the demineralized region of the scaffold. Previous studies have shown peroxide treatment to be effective in the removal of osteoinductivity. We first explored the effect of the exposure of demineralized bone to peroxide on the surface characteristics of the scaffolds by SEM. Demineralized bone has a multiscale pore structure that was unaffected by peroxide treatment (Figure 5). We also examined the effect of peroxide treatment on cell attachment and proliferation on the scaffolds (Figure 6). The ASC attachment to the peroxide-treated scaffolds was significantly less than attachment to the untreated scaffolds (*p* < .0001). Over time, proliferation proceeded as normal and was not inhibited by peroxide treatment. At day 5, there was no significant difference between the cells on the untreated and peroxide-treated scaffolds.

### Osteoinductivity assessment

To confirm that treatment with peroxide removes osteoinductive factors associated with demineralized bone, we measured ALP activity as an early marker for osteogenic differentiation and mineralization of whole scaffolds as an end-stage marker for differentiation (Figure 6). As expected, ASCs exposed to demineralized bone powder-treated with peroxide showed significantly less ALP activity after 7 days in culture than did ASCs exposed to untreated demineralized bone powder. Peroxide-treated demineralized bone powder evoked an ALP response similar in level to that of ASCs cultured in medium without any powder supplementation. Additionally, after 30 days in culture, μCT analysis did not reveal mineral deposition on the demineralized scaffolds, indicating that the demineralization treatment for this experiment lacked osteoinductive properties.

### Mechanical properties

To characterize the pre-implantation mechanical properties of the demineralized cancellous bone, long scaffolds treated with peroxide were loaded under uniaxial tension until failure. The stress-strain curves roughly followed the characteristic *J* shape of soft connective tissue. The tangent modulus, ultimate tensile strength (UTS) and the strain at failure were captured from the collected data. The tangent modulus was calculated as the slope of the linear section of the curve. Material properties of the scaffold, particularly modulus, were notably lower than those of normal ligaments and tendons. On average (*n* = 4 samples), the tangent modulus was 14.9 ± 8.8 MPa, while UTS and the strain at failure were measured at 1.23 ± 0.14 MPa and 0.29 ± 0.05 mm/mm, respectively. For comparison, the tangent modulus of the supraspinatus tendon in humans has a range of 40 to 170 MPa [35].

### Infraspinatus tendon repair

The postoperative period was without complications. The animals were ambulatory immediately post-surgery. No lameness was observed after the first 2 weeks. Animal recovery during the 16 weeks was unremarkable. Upon tendon retrieval, gross observation of the standard repaired tendons revealed a normal white tendinous appearance. The scaffold-treated specimens appeared slightly larger in thickness than standard-repaired tendons with a more notable redness on the medial surface of the tendon. Transection of the experimentally treated tendon displayed a highly vascularized tissue. Regenerated tissue showed solid integration with the soft connective tissue and the underlying bone, and there were no notable adhesions.

Histological examination did not reveal any inflammation in either control or experimental tendons. Standard repair-treated specimens revealed highly organized tendinous structure in the midsubstance of the host tissue. Dense connective tissue filled the defect located near the interface with random collagen organization as evidenced under polarized light microscopy (image not shown). In the interface region, periosteal bone formation was seen in the area below the insertion area, likely resulting from the disruption of the periosteum in the surgical procedure. A fibrocartilage region between the tendon and bone at the interface was not discernable in either toluidine blue- or tetrachrome-stained sections.

In specimens repaired with the tissue scaffold, a much more organized structure was found. Periosteal bone overgrowth was less evident in these samples. Newly deposited collagen fibers were found in the pore space of the soft tissue segment and appeared to be somewhat aligned in the loading direction. The fibers were integrated well with the older tendon tissue. Additionally, there was histological evidence of a normal tendon-to-bone interface only in the experimental samples. Tetrachrome staining revealed a complex heterogeneous transition from bone to calcified fibrocartilage, fibrocartilage, and tendon tissue. This region was slightly thicker and somewhat less organized than the normal enthesis.

### Supraspinatus tendon repair

Supraspinatus tendon explants appeared similar to the infraspinatus tendon explants in gross appearance. Both standard-treated and tissue scaffold-treated samples displayed a normal white tendinous structure. Scaffold-treated samples showed increased redness in the tendon midsubstance. In general, the size of the scaffold-treated tendon was larger than that of the standard-treated tendon. No adhesions to the surrounding tissue structures were noted. Palpation of the tendons did not reveal large ectopic bone formation within the soft tissue structure.

**Figure 7:**
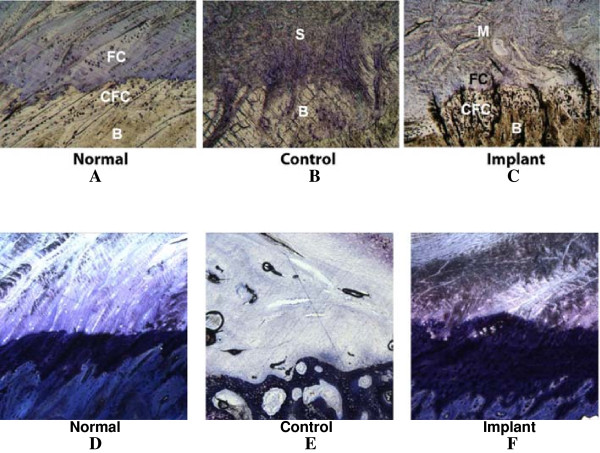
**Histological sections of the supraspinatus and infraspinatus tendons in sheep.** Histological sections of the supraspinatus (MacNeal's tetrachrome stain (**A**, **B**, and **C**)) and infraspinatus (toluidine blue stain (**D**, **E**, and **F**)) tendons in sheep demonstrate that the normal enthesis exhibits a typical bone-calcified fibrocartilage-fibrocartilage interface (**A**, **E**). The surgical control (**B**, **E**) transitions from bone directly into scar tissue in both tendons after 16 weeks. In contrast, the scaffold-treated supraspinatus tendon (**C**) demonstrates the regeneration of the bone-calcified fibrocartilage-fibrocartilage interface and a regrowth of tendon midsubstance, although the calcified fibrocartilage and fibrocartilage layers are thicker in the repair than in the normal animals. A similar response was observed in the scaffold-repaired infraspinatus tendon (**F**).

Histological evaluation of the control tendons demonstrated disorganized collagenous matrix with some evidence of local tendon thinning near the suture site. As with the infraspinatus tendon samples, periosteal bone formation was found in the repair site (Figure 7). The interface between the bone and the repaired tendon did not display a fibrocartilage layer. Instead, the new tendon tissue exhibited very little organization at the bone connection.

## Discussion

This study was designed to investigate the potential development of a biomaterial scaffold for the regeneration of orthopedic interfaces from natural biomaterials that provide the rapid transition from soft to hard tissue without compromising continuous load transfer pathways or nutrient transport. At 16 weeks postoperatively, scaffold-repaired tendons displayed histological evidence of a transitional zone from tendon to fibrocartilage to bone. While the repaired tendons exhibited thicker-than-normal transition regions, the transition was completely absent in tendons treated with standard primary repair. Instead, disorganized scar tissue filled the defect area between the tendon and the bone.

The use of cancellous bone as the raw material for this application is advantageous for a number of reasons. The scaffold resulting from regionally demineralized cancellous bone is a highly porous scaffold and contains all of the extracellular matrix (ECM) components of the orthopedic interface. This structure contains the basic region-specific extracellular matrix components of the natural orthopedic interface. The soft tissue region was comprised primarily of type I collagen, which is the primary component of the midsubstance of soft connective tissues, while the mineralized region retained the normal mineralized bone ECM. The ability of the ECM components to drive cellular proliferation, differentiation, and ECM production has been well established [27,36,37].

Peroxide treatment of the demineralized region of the orthopedic interface scaffold effectively removed osteoinductivity from this segment of the scaffold as revealed by differentiation assays performed using adult adipose-derived stem cells cultured with peroxide-treated and untreated demineralized bone. It was previously demonstrated that ALP activity of ASCs induced to undergo osteogenic differentiation peaks after culture for 7 days [33]. ASCs cultured with peroxide-treated demineralized bone expressed ALP activity equivalent to basal levels and did not produce mineral matrix in long-term culture. ASCs were chosen for this study with the rationale that if a large scale structure was needed, adipose tissue would be the easiest anatomic site from which to procure a stem cell source. These results generally agree with previous investigations using peroxide on demineralized bone [31,32]. Our findings suggest that osteoinductive factors present in demineralized bone are removed from the soft segment of the interface scaffold when exposed to peroxide.

Although peroxide is a powerful oxidizing agent, the concentration used here did not alter the microarchitecture of the demineralized segment of the scaffold interface as demonstrated by SEM imaging. While there was a slight decrease in cellular attachment to the peroxide-treated demineralized bone, the cytocompatibility of the demineralized bone did not appear to be compromised.

To our knowledge, this is the first study to report the development and tensile mechanical characterization of a naturally derived, porous scaffold for the regeneration of the orthopedic interface. In the few orthopedic interface regeneration studies performed, it is rare to find data on mechanical functionality, most likely due to the lack of continuity between the hard and soft segments [18]. With this interface scaffold, we found relatively low mechanical properties in tension. A substantial increase in the mechanical integrity of the scaffold will be necessary in order to withstand joint loads. Researchers have utilized crosslinking in the past to strengthen collagenous tissues, and we postulate that such methods will strengthen this orthopedic interface scaffold. Applicable approaches include physical crosslinking methods (e.g., dehydrothermal treatment and ultraviolet irradiation) and chemical crosslinking methods (e.g., carbodiimide and genipin). Using such techniques, it is also conceivable that the mechanical properties could be tailored specifically for a given application by altering crosslinking conditions, including length of time, temperature, and chemical reaction mixture concentrations, although the effects of crosslinking on the biological activity of cells must be determined. While crosslinking appears to be the best solution to ensure that the scaffold can withstand the requisite loading environment without affecting fluid transport through the structure, it may be possible to reinforce the scaffold using sutures, pins, or an internal removable bracing. In addition, the possibility that bone-inducing peptides may remain in the demineralized region has the potential to limit the applicability of the scaffold. While we used hydrogen peroxide to mitigate this effect, other methods to remove osteoinductivity, including heat and guanidine hydrochloride treatment, may be employed [38].

This preliminary investigation has several limitations. One limitation is the low number of animals used for the study. Absolute conclusions are difficult to make due to biological variability, and confirmation with more *in vivo* testing is necessary. However, a preliminary study was necessary before advancing to more intensive, exhaustive trials. A second limitation is that the study does not quantitatively assess the biochemical or biomechanical properties at the interface. While histology confirms success in the development of the structure of the orthopedic interface, further testing including macroscale mechanical tests, nanoindentation along the structure, and quantitative biochemical assessment are needed to confirm true regeneration. A longer-term study should also be conducted to determine whether or not the transition layer approaches the same thickness as that found in normal entheses. A third limitation of our study is the use of only an acute injury model, where healthy tissue was damaged and immediately repaired. A degenerative model will need to be developed for full applicability to rotator cuff defect repairs. This degenerative environment may not provide nutrients from the marrow and may require additional biochemical aids (e.g., platelet-rich plasma) to promote healing and regeneration. Despite these limitations, our results demonstrate substantial advantages over other proposed rotator cuff repair technologies. The scaffold described herein is a natural material and, as such, can be remodeled by the body over time to adapt to the loading environment of the joint. Unlike most other biomaterial repair options, the cancellous bone-derived scaffold has a built-in interface with direct load transfer between the hard and soft regions, which allows secure initial fixation to the bone. To date, only one other research group has attempted to develop a multiphase structure using PLGA and bioactive glass to simulate the junction between the hard and soft tissue [16,18]. However, while the manufacturing process produces a structure that is contiguous, the inherent discontinuities between the different phases may create stress concentrations at the microscale as load is transferred from one side to the other.

## Conclusion

In summary, our study has shown proof of concept for the use of a cancellous bone-derived scaffold for orthopedic interface regeneration in an ovine rotator cuff tendon model. The material integrated well with the host tissue, facilitated the development of organized collagenous tissue, reduced the formation of enthesophytes at the interface, and produced a four-zone fibrocartilagenous interface similar to the normal interface structure. Studies with a larger sample size and quantification of biochemical and biomechanical properties are needed to further validate these results. This scaffold represents a potential biomaterial solution for treatment of rotator cuff defects and other soft connective tissue injuries that require interface repair.

## Competing interests

The authors declare that they have no competing interests.

## Authors’ contributions

DAD and EAN conceived of the study. DADickerson manufactured the scaffolds and performed the *in vitro* assessments. GJB and TNM developed the experimental animal model and performed the surgeries. DCVS oversaw the histological analysis. All authors read and approved the final manuscript.
